# Development and validation of MIX: comprehensive free software for meta-analysis of causal research data

**DOI:** 10.1186/1471-2288-6-50

**Published:** 2006-10-13

**Authors:** Leon Bax, Ly-Mee Yu, Noriaki Ikeda, Harukazu Tsuruta, Karel GM Moons

**Affiliations:** 1 Julius Center for Health Sciences and Primary Care, UMC Utrecht, The Netherlands; 2 Department of Medical Informatics, Graduate School of Medical Sciences, Kitasato University, Japan; 3 Centre for Statistics in Medicine, Oxford, UK

## Abstract

**Background:**

Meta-analysis has become a well-known method for synthesis of quantitative data from previously conducted research in applied health sciences. So far, meta-analysis has been particularly useful in evaluating and comparing therapies and in assessing causes of disease. Consequently, the number of software packages that can perform meta-analysis has increased over the years. Unfortunately, it can take a substantial amount of time to get acquainted with some of these programs and most contain little or no interactive educational material. We set out to create and validate an easy-to-use and comprehensive meta-analysis package that would be simple enough programming-wise to remain available as a free download. We specifically aimed at students and researchers who are new to meta-analysis, with important parts of the development oriented towards creating internal interactive tutoring tools and designing features that would facilitate usage of the software as a companion to existing books on meta-analysis.

**Results:**

We took an unconventional approach and created a program that uses Excel as a calculation and programming platform. The main programming language was Visual Basic, as implemented in Visual Basic 6 and Visual Basic for Applications in Excel 2000 and higher. The development took approximately two years and resulted in the 'MIX' program, which can be downloaded from the program's website free of charge. Next, we set out to validate the MIX output with two major software packages as reference standards, namely STATA (metan, metabias, and metatrim) and Comprehensive Meta-Analysis Version 2. Eight meta-analyses that had been published in major journals were used as data sources. All numerical and graphical results from analyses with MIX were identical to their counterparts in STATA and CMA. The MIX program distinguishes itself from most other programs by the extensive graphical output, the click-and-go (Excel) interface, and the educational features.

**Conclusion:**

The MIX program is a valid tool for performing meta-analysis and may be particularly useful in educational environments. It can be downloaded free of charge via  or .

## Background

The amount of data produced by researchers in health sciences has been growing explosively and advances in genetics, genomics, and information technology are likely to further contribute to this growth. In the past two decades, meta-analysis has evolved into the statistical method par excellence to make sense out of the growing number of research reports. As the quantitative analytical part of a systematic review, it has been used for evaluating data from both experimental and observational studies in therapeutic, diagnostic, prognostic, and etiologic settings. In the commonly used definition of the hierarchy of scientific data for medical decision making, meta-analyses are considered as providing the highest level of evidence [1,2]. As such, they can have a major impact on medical practice and health care policies, especially if aggregating data and investigating sources of heterogeneity provide new insights. Two well-known examples are the meta-analyses by Yusuf et al [3] and Lau et al [4], both showing that meta-analysis can be a powerful tool to show intervention effects that would remain beneath the surface of single study data without proper synthesis and re-analysis.

**Figure 1:**
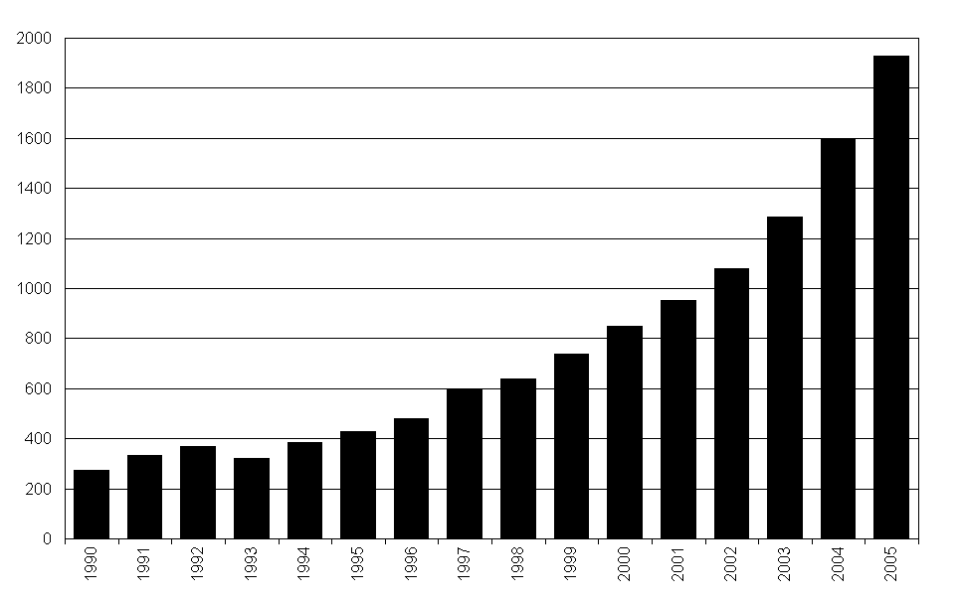
**The annual number of meta-analyses registered by PubMed**. An overview of studies of the publication type "meta-analysis" from 1990 to 2005 in PubMed.

Although meta-analyses can be applied to all types of medical research, its primary application so far has been in the therapeutic realm. One of the main forces behind the rise of therapeutic meta-analysis is the Cochrane Collaboration [5], whose effort to systematically assess and synthesize evidence from randomized controlled trials has so far produced more than 4400 Cochrane systematic reviews, many with quantitative meta-analyses. The increasing interest for meta-analysis in health sciences over the past twenty years has been reported by several authors [6-11] and a small search we did in preparation of this project reveals that between 1990 and 2005 approximately 12,000 publications have been classified as a meta-analysis by PubMed. A bar graph of the annual numbers suggests that the interest for meta-analysis is still increasing (figure 1).

Many general statistical software packages have included options for meta-analysis in their basic program configuration, and user-communities have written numerous meta-analysis add-ons. Specialized software packages, meant exclusively for meta-analysis, are also available in various types and price ranges. Although the number of software packages for performing meta-analysis is substantial, in our opinion, most share one common limitation: low applicability in educational settings or environments with beginning researchers. Even though numerous researchers in health care are nowadays confronted with data from published meta-analyses or are even requested to do a meta-analysis themselves, there is still little or no electronic educational material and none of the existing software has explicit educational features. Cost is another issue that may have an impact on the use of software by students and lecturers: only a few of the modern meta-analysis packages are free and if academic pricing is available, prices can still be rather high for many.

After reading previously published software reviews [12-15] and using existing meta-analysis software, we made an inventory of what we thought was lacking or could be improved. Next, we set out to implement our ideas and create an innovative and comprehensive statistical meta-analysis package that would be freely accessible and user-friendly enough for students and beginning researchers. The program, called MIX (Meta-analysis with Interactive eXplanations), has been developed over the past two years and has been presented at several stages of the development at a number of conferences [16-19]. In October 2005, the first public version (1.0) was released during the Cochrane Colloquium in Melbourne [19] and has become available for download via the MIX website [20]. It has been receiving a lot of interest (100–150 unique visitors to the MIX website each week) and has been downloaded over 1800 times within 6 months of its first release. This has prompted us to validate the results of all tests in the program formally and this article provides the offcial introduction of the MIX program together with the results of the validation.

## Implementation

### Objectives

Our primary objective was to develop a free program for meta-analysis of causal research (therapeutic trials as well as etiologic cohorts and case-control studies) that could be applied in both analytical and educational settings. Our secondary aim was to validate the analytical tests in the program with output from established reference standards.

### Program development

Before the actual development, we started with making an inventory of the most important meta-analytical tests and approaches, and brainstormed on ideas for an interface. Since causal meta-analysis methods are relatively well-established (in contrast to diagnostic or prognostic approaches to meta-analysis), we focused on meta-analysis of controlled trials and cohort or case-control studies. In these studies, outcome differences between exposed or treated and non-exposed or untreated groups are compared to assess a causal relationship between the determinant (treatment or exposure) and an outcome (mortality or morbidity). As far as the program structure was concerned, our a priori idea was to create an add-in for Excel. Although a rather unorthodox approach in this area (all existing meta-analysis programs are stand-alone programs and work independently of Microsoft Office), Excel provides a sophisticated calculation and graphics platform that is well-suited to many meta-analytical methods and at the programmer's disposal before any programming is done. Consequently, development and maintenance is relatively easy and costs can be kept to a minimum (one of the main aims in our program development). Furthermore, the spreadsheet environment of Microsoft Excel is familiar to almost all researchers in medical, social, and economical sciences, which was very much in line with our attempt to develop a package that is fit for beginning researchers. Although we realized that even recent versions of Excel can be inaccurate with regard to some statistical calculations [21-23], we were confident that we could program around these difficulties if necessary.

Since we wanted to move beyond the occasional spreadsheet that can perform meta-analytical calculations, we started by designing a programming structure in which the already existing Excel functionality could be exploited to its maximum. Sophisticated procedures were custom-programmed with Visual Basic in the Visual Basic for Applications (VBA) editor of Excel 2003 (and tested in Excel 2000 and onward). The so-called front-loader (a start-up program initiated with an icon) and some small assistant programs, all being non-Excel entities, were developed with Visual Basic 6.0 (VB6).

### Program architecture and operation

The current version of the program (version 1.5) is still only compatible with Windows operating systems running Excel 2000 or later, but versions for use with Excel on Macintosh and Linux are in preparation. The descriptions below apply to the Windows version, though most of it can be extended to future versions for other operating systems.

**Figure 2:**
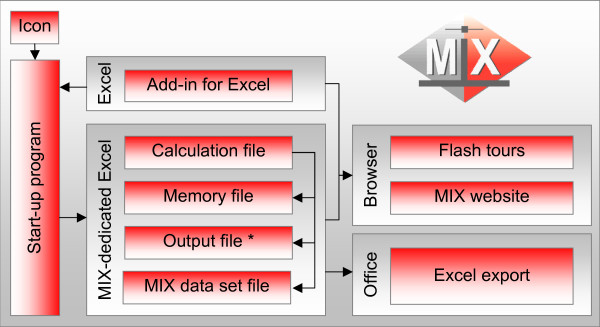
**Structure of the MIX program**. The MIX program is started by simply clicking the MIX icon on the desktop or in the Windows Start Menu. The program uses a number of Excel workbooks, of which only the output file (*) is directly accessible by the user. Via the custom interface, several educational features can be accessed and custom meta-analysis reports can be produced.

Installation is made easy with a set-up program that installs the necessary files in a folder that can be specified by the user (default is C:\Program Files\MIX). It will also create a MIX item in the Windows Start Menu (installing additional start-up icons on the Desktop or in the Quick-Launch bar is optional) and provides the option to start a Flash^®^-based program introduction. The MIX menu item contains an icon for starting up the MIX program, a folder with a shortcut to the uninstall program, a folder with shortcuts to programs for loading and unloading the Excel add-in, and a folder with educational programs and information. Loading the small MIX add-in that is supplied with the main program (typically automatically loaded during installation) results in a MIX menu-item under the Tools menu in Excel. This MIX menu contains several functions that can be accessed when the MIX program itself is not running. The files that form the core of the program are recognizable by their Mix file extension (*.mix) and currently contain approximately 16,000 lines of command code in 26 code modules and 17 custom user forms. These core files take up approximately 22 Mb of space on a hard-disk and their primary functions are (A) running interface procedures, (B) showing and manipulating output, (C) performing analyses, and finally (D) exporting and communicating with external files and programs. One of the core files is a large Excel workbook with 23 worksheets that forms the calculation engine of the program. It contains 6 sheets with primarily worksheet formulas and 10 sheets with various kinds of pre-calculated graphical and numerical results from meta-analytical tests. The remaining sheets contain information for help functions or programming purposes. This Excel workbook remains hidden from the users at all times. Figure 2 gives a graphical representation of the full program structure.

At start-up, a dedicated instance (an independent fully functional running program) of Excel is created and becomes visible once all regular Excel menus and toolbars are hidden and replaced by the MIX graphical interface. The Excel instance used by MIX is secured for exclusive use by the MIX program and does not interfere with existing Excel windows or settings.

**Figure 3:**
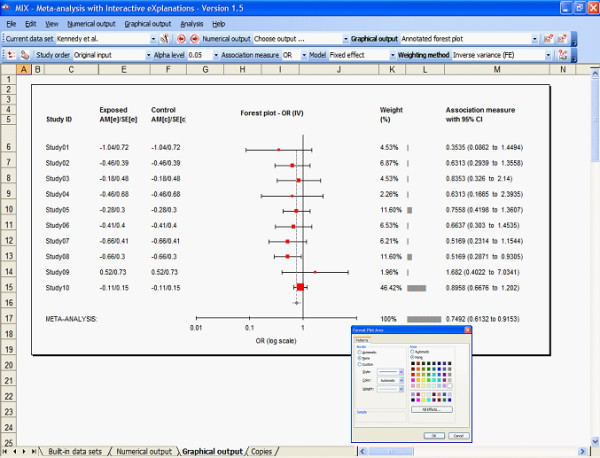
**The MIX program's graphical interface with a forest plot**. The standard Excel menu and toolbars have been replaced by the MIX interface through which graphical and numerical output can be created and manipulated. Custom shortcut menus are available via right-clicks and double clicking graphical items shows the formatting options that Excel users are familiar with.

The interface consists of a menu bar, two toolbars, and several shortcut menus. The menu bar and toolbar are directly accessible and the shortcut menus pop up with a right click of the mouse. The MIX menu bar has eight main menus (File, Edit, View, Numerical Output, Graphical Output, Analysis, and Help) via which all functions of the MIX program can be executed. Most of the common functions require only a single click on the toolbars. Double clicking graph items skips the shortcut menu and directly provides options for changing the graph item's format. Figure 3 shows the MIX program's user-interface with a forest plot and a format box to change the graph's format.

**Figure 4:**
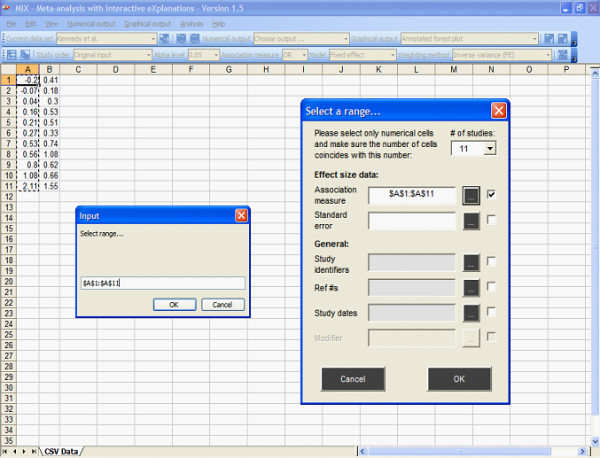
**Creation of a data set with the MIX program**. Data sets can be created from Excel files, Comma Separated Value (CSV) files, or via manual input. Once the data prepared on a spreadsheet within the program, the user can select the cell ranges that correspond to the relevant variables and load the data for analysis with a simple click.

The MIX program provides several options for importing or creating data sets for meta-analysis. The most convenient option is to create an Excel or CSV file with data (standard output option in Excel) and import this file into the MIX program. The variable ranges are then selected in Excel-manner to create a data set (see figure 4), which is subsequently loaded for analysis and optionally saved as a MIX data set file (*.mxd). The program accepts descriptive data from studies with continuous outcomes, e.g. sample size, mean, standard deviation, and dichotomous outcomes, e.g. group sizes and event numbers (two-by-two table data). Comparative data can also be loaded by means of association measures with their standard error. Initially, however, it is not necessary to make a data set since 19 data sets from the most authoritative books on the subject ("Meta-analysis in Medical Research" by Sutton et al [10], "Systematic Reviews in Health Care, Meta-Analysis in Context" by Egger et al [6], and Systematic Reviews in Health Care, A Practial Guide by Glasziou et al [7]) have been included in the program. Most analyses and graphs presented in these books can be reproduced with a few clicks and the program can be used as a learning or teaching companion to these books. We hope to support more more books in this way in the future. In addition, the MIX website also contains a data set repository where users can contribute and download MIX data sets.

A large variety of numerical and graphical output can be produced by the program. Besides the association measure values from the meta-analysis, several formal tests for heterogeneity, small study effects (publication bias), single study influence, and cumulative trends are also available in MIX. The graphical output is particularly comprehensive, with no less than eighteen informative plots that can be formatted in detail.

Possible association measures from continuous outcome data input are mean difference (MD), Hedges' g (HG), and Cohen's d (CD), analyzed by inverse variance fixed or random effects models. Data from studies with dichotomous outcomes can be analyzed with a risk difference (RD), risk ratio (RR), or odds ratio (OR), weighted by inverse variance, Mantel-Haenszel, Peto (only odds ratio), or Dersimonian-Laird approaches. Analyses based on correlation coefficients or Fisher's Z are also possible, though only if the data are provided as comparative input, e.g. the association measures itself with their standard error. If correlation or effect size data are not in this format, they can be transformed via the MIX Statistics Converter that comes with the program. Table 1 gives an overview of the general features and the numerical and graphical methods in version 1.5 of the MIX program.

**Table 1:** Overview of the MIX program's features

**General**	**Input options**
- Program size: 22 Mb (with 20 Mb tutor files)	- Descriptive dichotomous (2 × 2 table data)
- Compatibility: Windows, Excel 2000 or later	- Descriptive continuous (n, m sd)
- Installation: Standard set-up program	- Comparative (am, se)
	- Max 100 studies
**Numerical output options**	
- Outcome measures	**Data input options**
◦ Odds ratio	- Manual
◦ Risk ratio	- Excel and CSV-files
◦ Risk difference	- Effect size and statistic conversions
◦ Mean difference	
◦ Hedges' g, Cohen's D	**Graphical output options**
◦ Correlation coefficient, Fisher's Z	- Box-and-whiskers plots
	- Z-score histogram
- Fixed effect analyses	- Normal-quantile plot
◦ Inverse variance	- Standard & annotated forest plots
◦ Mantel-Haenszel	◦ Points proportional to study weights
◦ Peto	- Cumulative forest plot
- Random effects analyses	- Galbraith plot
◦ Dersimonian-Laird	◦ Radial axis
- Cumulative analyses	- Exclusion sensitivity plot
◦ Ordered by publication date	- L'Abbe plot
◦ Ordered by other variables	- Baujat plot
- Individual study data	- Funnel plot
◦ Outcome results	◦ 1/se, se, P value, N
◦ P-values	- Egger's regression plot
◦ Weights	- Macaskill's regression plot
	- Trim-and-fill plot
- Heterogeneity	
◦ Cochran's Q	**Export options**
◦ Higgins' H and I^2^	- Output to clipboard
	- Data set to Excel or CSV
- Publication bias	- Ready-made reports to Excel
◦ Fail-safe N	- Multi-resolution graphs to clipboard
◦ Begg's rank correlation test	
◦ Egger's regression test	**Learning items**
◦ Macaskill's regression test	- Output tutor
◦ Trim-and-fill test	- Concept tutor
- Continuity correction	- Built-in data sets
◦ User-defined value	- Theory guide (Flash)
◦ C~1/n opposite group	- Program guide (Flash)

The overview gives a general summary of the features in version 1.5 of the MIX program. More details are provided on the website. Abbreviations: "n" = group size, "m"' = mean, "sd" = standard deviation, "am" = association measure, se" = standard error, "N" = sample size.

The most important educational features are the program's Output Tutor and Concept Tutor. Both are interactive dialog boxes that provide information about epidemiological and statistical concepts and tests. The Output Tutor changes with each analysis and always explains tests and results that are displayed or changed at the very moment. Additional teaching material includes a Flash^®^-based Theory Tour that explains the fundamentals of systematic reviews and meta-analyses and a Program Tour that shows the basics of how to use the program. The educational materials take up approximately 25 Mb and can also be downloaded separately.

To increase program stability and prevent users from accidentally altering the Visual Basic procedures, the source code cannot be accessed while the program is running. Codes to unlock the VBA modules are provided by the first author upon request.

### Validation

Version 9.2 of STATA [24], and more specifically version 1.81 of the *metan *program [25], version 1.2.4 of the *metabias *program [26], and version 1.0.5 of the *metatrim *program [27] were used as the general reference standards for most tests. Details on the development of these user-written programs themselves can be found in the STATA Technical Bulletins [25-27]. The meta-analysis software Comprehensive Meta-Analysis (CMA) version 2 [28] was used for validation of the Fail-safe N output and to double check the results of the other tests. Two investigators (LB, LMY) performed the validation independently with the MIX program (version 1.5 running in Excel 2003) and the reference standard(s) by analyzing eight data sets from meta-analyses that have been published in major journals [4,29-35].

The data sets represent three of the most often used types of data for meta-analysis in health care research: 1) descriptive data for dichotomous outcomes, 2) descriptive data for continuous outcomes, and 3) comparative (association measure) data. For all three data types we chose a relatively small (less than 10 studies) and large data set (more than 20 studies) and we used two extra data sets in the 'descriptive dichotomous' category (one representing a meta-analysis of substantially heterogeneous studies and one with a rare event). The data sets are summarized in table 2. The tests that were subject to the validation procedures are shown in table 3. The items include individual study association measures, combined association measures, and several heterogeneity and small study effect assessments. Whenever applicable, p-values and/or confidence intervals were also compared.

**Table 2:** Overview of the data sets used in the validation

**#**	**Author(s)**	**Date**	**Studies**	**Input type**
1	Lau et al. [4]	1992	33	DD – large
2	Hodnett et al. [29]	2001	5	DD – small
3	Teo et al. [30]	1991	16	DD – publication bias
4	Crowley [31]	2000	17	DD – rare events
5	Lightowler et al. [32]	2003	5	DC – small
6	Wahlbeck et al. [33]	2000	11	DC – medium large
7	Pagliaro et al. [34]	1992	19	C – odds ratio
8	Law et al. [35]	1994	10	C – risk difference

The validation was done with eight data sets from meta-analysis that have been published in major peer-reviewed journals. The data sets were selected to represent a wide spectrum of potential input for meta-analysis. Abbreviations: "DD" = descriptive data for dichotomous outcomes (two-by-two table data), "DC" = descriptive data for continuous outcomes (means with their standard deviations and sample sizes), and "C" = comparative data (association measures with standards error or confidence intervals).

**Table 3:** Meta-analytical tests that were part of the validation

**Study data (per association measure/weighting)**
- Association measure with 95% CI and/or P value
- Study contribution weights
**Meta-analysis (per association measure/weighting)**
- Association measure with 95% CI and P value
- Heterogeneity Q with 95% CI and/or P value
- Inconsistency I^2 ^with 95% CI and/or P value
- Fail-safe N with tolerance level
- Begg's rank correlation test with z-score and P value
- Egger's regression intercept with 95% CI and/or P value
- Macaskill's regression slope with 95% CI and/or P value
- Trim-and-fill studies with new association measure and 95% CI

Essentially all major numerical output that is produced by a comprehensive meta-analysis was assessed during the validation. The tests were repeated with all available (fixed effect and random effects) weighting models. Abbreviations: "CI" = confidence interval.

Results from the analyses of the eight data sets with MIX and the reference software were entered independently in identical custom-made spreadsheets. These spreadsheets were later compared in separate analysis sheets that used a cell-based formula to check for discrepancies of results up to 4 decimals.

## Results and Discussion

In summary, we have been able to achieve our objective of developing a comprehensive and yet free program for meta-analysis. The Excel platform, although not without problems, has proved to be flexible enough to create an easy-to-use, and graphically and numerically comprehensive program.

In its current state (version 1.5) all results from the MIX program are identical (up to 4 decimals minimally) to results from the most recent versions of the *metan*, *metabias*, and *metatrim *commands in STATA. The small study effect regression test by Macaskill [36] that was tested via STATA's *regress *command also turned out to be accurate. Table 4 and 5 are examples of the odds ratio validation results for data set 1 [4].

**Table 4:** Individual study weighting validation with data set 1

	**Weighting method and weights (%)**							
	
	**IV**		**MH**		**PETO**		**IV+t**	
**Studies**	**MIX**	**STATA**	**MIX**	**STATA**	**MIX**	**STATA**	**MIX**	**STATA**

Fletcher	0.07%	0.07%	0.18%	0.18%	0.11%	0.11%	0.21%	0.21%
Dewar	0.21%	0.21%	0.27%	0.27%	0.22%	0.22%	0.59%	0.59%
Euro 1	0.74%	0.74%	0.53%	0.53%	0.74%	0.74%	1.99%	1.99%
Euro 2	3.38%	3.38%	3.68%	3.68%	3.39%	3.39%	7.18%	7.18%
Heikinheimo	0.95%	0.95%	0.74%	0.74%	0.95%	0.95%	2.50%	2.50%
Italian	0.89%	0.89%	0.76%	0.76%	0.88%	0.88%	2.35%	2.35%
Australia 1	1.39%	1.39%	1.39%	1.39%	1.38%	1.38%	3.50%	3.50%
Frankfurt	0.80%	0.80%	1.18%	1.18%	0.90%	0.90%	2.14%	2.14%
NHLBI-SMIT	0.21%	0.21%	0.12%	0.12%	0.24%	0.24%	0.60%	0.60%
Frank	0.29%	0.29%	0.26%	0.26%	0.29%	0.29%	0.82%	0.82%
Valere	0.42%	0.42%	0.35%	0.35%	0.42%	0.42%	1.17%	1.17%
Klein	0.07%	0.07%	0.04%	0.04%	0.10%	0.10%	0.21%	0.21%
UK-Collab	1.85%	1.85%	1.67%	1.67%	1.81%	1.81%	4.46%	4.46%
Austrian	2.23%	2.23%	2.64%	2.64%	2.34%	2.34%	5.21%	5.21%
Australia 2	1.14%	1.14%	1.24%	1.24%	1.13%	1.13%	2.94%	2.94%
Lasierra	0.07%	0.07%	0.14%	0.14%	0.09%	0.09%	0.20%	0.20%
N Ger Collab	2.35%	2.35%	1.85%	1.85%	2.33%	2.33%	5.44%	5.44%
Witchitz	0.22%	0.22%	0.22%	0.22%	0.22%	0.22%	0.64%	0.64%
Euro 3	1.05%	1.05%	1.24%	1.24%	1.09%	1.09%	2.73%	2.73%
ISAM	2.96%	2.96%	2.74%	2.74%	2.92%	2.92%	6.50%	6.50%
GISSI-1	33.05%	33.05%	31.84%	31.84%	32.65%	32.65%	21.00%	21.00%
Olson	0.07%	0.07%	0.10%	0.10%	0.08%	0.08%	0.20%	0.20%
Baroffio	0.05%	0.05%	0.30%	0.30%	0.15%	0.15%	0.14%	0.14%
Schreiber	0.08%	0.08%	0.13%	0.13%	0.10%	0.10%	0.22%	0.22%
Cribier	0.05%	0.05%	0.04%	0.04%	0.05%	0.05%	0.15%	0.15%
Sainsous	0.20%	0.20%	0.26%	0.26%	0.22%	0.22%	0.57%	0.57%
Durand	0.17%	0.17%	0.19%	0.19%	0.17%	0.17%	0.47%	0.47%
White	0.18%	0.18%	0.54%	0.54%	0.35%	0.35%	0.51%	0.51%
Bassand	0.25%	0.25%	0.30%	0.30%	0.27%	0.27%	0.71%	0.71%
Vlay	0.06%	0.06%	0.09%	0.09%	0.07%	0.07%	0.19%	0.19%
Kennedy	0.71%	0.71%	0.78%	0.78%	0.71%	0.71%	1.90%	1.90%
ISIS-2	43.68%	43.68%	43.92%	43.92%	43.47%	43.47%	22.18%	22.18%
Wisenberg	0.14%	0.14%	0.28%	0.28%	0.16%	0.16%	0.40%	0.40%

In a meta-analysis, each study is given a weight that determines its influence on the overall result and this weight depends on the weighting method. Proper weighting is crucial to get correct results, so we validated all individual study weights for each data set and weighting method. The table shows the odds ratio weighting validation for data set 1. Abbreviations: "IV" = inverse variance weighting, "MH" = Mantel-Haenszel weighting, "PETO" = Peto weighting, and "IV+t" = inverse variance plus tau, which refers to random effects weighting according to the DerSimonian-Laird method.

**Table 5:** Summary of the validation with data set 1

	**MIX**	STATA/CMA *
	
**Items in odds ratio meta-analysis**	**Results**	**Results**
**Fixed effect (IV) odds ratio**		
OR – IV (95% CI)	0.7677 (0.7196 to 0.8190)	0.7677 (0.7196 to 0.8190)
Z (P value)	8.0073 (< 0.0001)	8.01 (< 0.0001)
Q (P value)	39.48 (0.17)	39.48 (0.17)
I^2 ^(95% CI)	0.1895 (0 to 0.4749)	0.1895
		
Fail-safe N (tolerance level)	270 (175)	270 *
Rank correlation tau ** (P value)	0.1799 (0.141)	(0.141)
Egger's regression intercept (95% CI)	-0.2955 (-0.7880 to 0.1970)	-0.2955 (-0.7880 to 0.1970)
Macaskill's regression slope (P value)	0 (0.8396)	0 (0.840)
Trim-and-fill association measure (95% CI)	0.7744 (0.7260 to 0.8260)	0.774 (0.726 to 0.826)
Trim-and-fill imputed studies	6	6
		
**Random effects (IV + t) odds ratio**		
OR – IV + t (95% CI)	0.7619 (0.6825 to 0.8506)	0.7619 (0.6825 to 0.8506)
Z (P value)	-4.84 (< 0.0001)	-4.84 (< 0.0001)
t^2^	0.117	0.117
		
Trim-and-fill association measure (95% CI)	0.7828 (0.6907 to 0.8871)	0.783 (0.691 to 0.887)
Trim-and-fill imputed studies	6	6

This table shows the odds ratio validation of the inverse variance fixed effect and random effects analyses with data set 1. Though not shown here, the results from Mantel-Haenszel and Peto weighting were evaluated similarly and so were the other association measures (risk difference and risk ratio). The process was repeated for each data set. (*): Results marked with '*' were produced by CMA. (**): The rank correlation tau is a continuity corrected tau-b. Abbreviations: "IV" = inverse variance weighting, "OR" = odds ratio, "CI" = confidence interval, "IV+t" = inverse variance plus tau, which refers to random effects weighting according to the DerSimonian-Laird method.

With regard to the trim-and-fill analysis [37], the MIX program allows for calculations using the weighting method applied in the original meta-analysis, whereas both CMA and STATA use only fixed or random effects inverse variance methods when trimming and filling. While the calculations in MIX for trim-and-fill analyses with other weighting methods were verified manually and we have no reason to believe anything is wrong, we recommend using the inverse variance methods until more is known about approaches with alternative weighting.

Although we are in the process of completing a formal software comparison project, we are confident that the MIX program can compete in many respects (usability, analytical options, comprehensiveness, and export options) with most of the existing meta-analysis programs like Comprehensive Meta-Analysis [28], MetaWin [38], RevMan [39], or WEasyMA [40]. However, there are also still some limitations. One is the maximum number of studies that can be analyzed in the meta-analysis, which is now 100. Though systematic reviews finding 100 studies for analysis are still very rare, this is something that may change in the future. Furthermore, while sub-group analyses are easy to perform within MIX, they are currently not automated and during a sub-group analysis not all subgroups can be shown simultaneously in a single forest plot. The subgroup forest plot can however be created manually because the Excel graphs of individual forest plots are relatively easily formatted and stacked. We intend to improve the program with regard to these limitations in the near future.

Another important issue that we will focus on in upcoming updates is meta-regression. Although some univariable regression methods are integrated in the tests for small study effects, the MIX program can currently not perform meta-regression. We realize that meta-regression, especially with multiple independent variables, is a valuable tool for assessing heterogeneity and adapting a meta-analysis accordingly, but it requires matrix calculations that are far more difficult to program in Excel or VBA than the standard tests. Currently, univariable meta-regression is possible with Comprehensive Meta-Analysis [28] and MetaWin [38]. However, like all dedicated meta-analysis packages they lack the option for multivariable meta-regression. We have started working on facilities for meta-regression within the MIX program and we hope it will be integrated sometime in 2007.

Finally, because we are still frequently updating the program and including new features, we have postponed the making of a hard-copy manual or methods guide until this process has stabilized.

## Conclusion

The MIX program provides researchers, students, and lecturers with a free tool to perform state-of-the-art meta-analyses and learn or teach about what it is they are doing. It uses an innovative approach with Excel as a computing platform and even provides some numerical and graphical output that is not provided by other software. Results from version 1.5 of the MIX program are identical to those from STATA, and MIX can be regarded as a comprehensive and valid tool for performing causal meta-analyses.

## Availability and requirements

Project name: MIX

Project homepage:  or 

Operating system(s): Microsoft Windows

Programming language: Visual Basic (VB6, VBA)

Other requirements: Microsoft Excel 2000 or later

License: Open Source, free

## Competing interests

The author(s) declare that they have no competing interests.

## Authors' contributions

LB designed and developed the MIX program, under supervision of NI and HT and with testing by and recommendations from all authors. The validation was performed by LB and LY and supervised by KGM. LB drafted the manuscript and all authors participated in the writing.

## Pre-publication history

The pre-publication history for this paper can be accessed here:



## References

[B1] Oxford-Center for Evidence Based Medicine, Levels of Evidence and Grades of Recommendation. http://www.cebm.net/levels_of_evidence.asp.

[B2] Yusuf S, Cairns J, Camm A, Fallen E, Gersh B (1998). Evidence-Based Cardiology.

[B3] Yusuf S, Zucker D, Peduzzi P, Fisher L, Takaro T, Kennedy J, Davis K, Killip T, Passamani E, Norris R (1994). Effect of coronary artery bypass graft surgery on survival: overview of 10-year results from randomised trials by the Coronary Artery Bypass Graft Surgery Trialists Collaboration. Lancet.

[B4] Lau J, Antman E, Jimenez-Silva J, Kupelnick B, Mosteller F, Chalmers T (1992). Cumulative meta-analysis of therapeutic trials for myocardial infarction. N Engl J Med.

[B5] The Cochrane Collaboration. http://www.cochrane.org.

[B6] Egger M, Davey Smith G, Altman D (2001). Systematic Reviews in Health Care: Meta-Analysis in Context.

[B7] Glasziou P, Irwig L, Bain C, Colditz G (2001). Systematic Reviews in Health Care: A Practical Guide.

[B8] Petitti D (2000). Meta-Analysis, Decision Analysis, and Cost-Effectiveness Analysis: Methods for Quantitative Synthesis in Medicine.

[B9] Stangle D, Berry D (2000). Meta-analysis in Medicine and Health Policy.

[B10] Sutton A, Abrams K, Jones D, Sheldon T, Song F (2000). Methods for Meta-Analysis in Medical Research.

[B11] Whitehead A (2002). Meta-Analysis of Controlled Clinical Trials.

[B12] Egger M, Sterne J, Smith G (1998). Meta-analysis software. BMJ.

[B13] Normand S (1995). Meta-analysis software – a comparative review – DSTAT, version 1.10. Am Statistician.

[B14] Sterne J, Egger M, Sutton A, Egger M, Davey Smith G, Altman D (2001). Meta-analysis software. Systematic Reviews in Health Care: Meta-Analysis in Context.

[B15] Sutton A, Lambert P, Hellmich M, Abrams K, Jones D, Berry D, Stangl D (2000). Meta-analysis in practice: A critical review of available software. Meta-Analysis in Medicine and Health Policy.

[B16] Bax L, Ikeda N, Shirataka M, Takeuchi A (2004). Explaining common meta-analytic statistics in Japan with a simple Excel add-in. The 24th Joint Conference on Medical Informatics.

[B17] Bax L, Ikeda N (2004). Explaining and performing common meta-analytic procedures in Japan: development of bilingual interactive software. The 12th Cochrane Colloquium.

[B18] Bax L, Tsuruta H, Ikeda N, Takeuchi A, Shirataka M (2005). The MIX program, free software for learning, teaching, and exploring meta-analysis with Excel. The 13th Cochrane Colloquium, Melbourne, Australia.

[B19] Bax L, Tsuruta H, Shirataka M, Takeuchi A, Ikeda N (2005). The MIX program, an active way of learning about meta-analysis with Excel. International Symposium: Systematic Review and Meta-Analysis, Wako, Japan.

[B20] Meta-analysis with Interactive explanations. http://www.mix-for-meta-analysis.info.

[B21] Knusel L (2005). On the accuracy of statistical distributions in Microsoft Excel 2003. Comput Statist Data Anal.

[B22] McCullough B, Wilson B (2002). On the accuracy of statistical procedures in Microsoft Excel 2000 and Excel XP. Comput Statist Data Anal.

[B23] McCullough B, Wilson B (2005). On the accuracy of statistical procedures in Microsoft Excel 2003. Comput Statist Data Anal.

[B24] StataCorp (2005). Stata Statistical Software, Release 9.

[B25] Bradburn M, Deeks J, Altman D (2003). Metan – an alternative meta-analysis command (Metan 1.81). Stata Technical Bulletin.

[B26] Steichen T (2003). Tests for publication bias in meta-analysis (Metabias 1.2.4). Stata Journal.

[B27] Steichen T (2003). Nonparametric trim and fill analysis of publication bias in meta-analysis (Metatrim 1.0.5). Stata Technical Bulletin.

[B28] Borenstein M, Hedges L, Higgins J, Rothstein H (2005). Comprehensive Meta-Analysis Version 2.

[B29] Hodnett E (2000). Caregiver support for women during childbirth. Cochrane Database Syst Rev.

[B30] Teo K, Yusuf S, Collins R, Held P, Peto R (1991). Effects of intravenous magnesium in suspected acute myocardial infarction: overview of randomised trials. Bmj.

[B31] Crowley P (2000). Interventions for preventing or improving the outcome of delivery at or beyond term. Cochrane Database Syst Rev.

[B32] Lightowler J, Wedzicha J, Elliott M, Ram F (2003). Non-invasive positive pressure ventilation to treat respiratory failure resulting from exacerbations of chronic obstructive pulmonary disease: Cochrane systematic review and meta-analysis. Bmj.

[B33] Wahlbeck K, Cheine M, Essali M (2000). Clozapine versus typical neuroleptic medication for schizophrenia. Cochrane Database Syst Rev.

[B34] Pagliaro L, D'Amico G, Sorensen T, Lebrec D, Burroughs A, Morabito A, Tine F, Politi F, Traina M (1992). Prevention of first bleeding in cirrhosis. A meta-analysis of randomized trials of nonsurgical treatment. Ann Intern Med.

[B35] Law M, Wald N, Thompson S (1994). By how much and how quickly does reduction in serum cholesterol concentration lower risk of ischaemic heart disease?. Bmj.

[B36] Macaskill P, Walter S, Irwig L (2001). A comparison of methods to detect publication bias in meta-analysis. Stat Med.

[B37] Duval S, Tweedie R (2000). Trim and fill: A simple funnel-plot-based method of testing and adjusting for publication bias in meta-analysis. Biometrics.

[B38] Rosenberg M, Adams D, Gurevitch J (2000). MetaWin: Statistical Software for Meta-Analysis Version 2.

[B39] The Nordic Cochrane Centre (2003). Review Manager (RevMan) Version 42 for Windows.

[B40] Chevarier P, Cucherat M, Freiburger T, Maupas J, Visele N, Bugnard F, Bazog P (2000). WeasyMA.

